# 

**Published:** 1872-02-01

**Authors:** 


Money Receipts to February 1, 1872.—Drs. J. H. Judson, $3.00; W. J. Fairman, $6.00; E. E. Kendell, $3.00; S. W. Ranson, $6.00; E. Holdness, $5.00; B. McMannamy, $3.00; C. D. Watson, $3.00; C. N. Cooper, $3.00; H. N. Hurlbut, $3.00; Flood, $3.00; W. H. Cook, $3.00; J. Anderson, $3.00; R. O. Phillips, $3.00; H. A. Hicks, $1.50; T. G. Williams, $3.00; W. W. Grant, $3.00; Jos. Hayton, $3.00; G. O. Bardwell, $3.00; D. L. Brookhart, $6.00; S. Rex Bucher, $3.00.

